# Meta-analysis of risk factors associated with suicidal ideation after stroke

**DOI:** 10.1186/s12991-021-00378-8

**Published:** 2022-01-05

**Authors:** Shuangmei Zhang, Anrong Wang, Weifeng Zhu, Zhaoyang Qiu, Zhaoxu Zhang

**Affiliations:** 1 Department of Rehabilitation, Cancer Hospital of the University of Chinese Academy of Sciences (Zhejiang Cancer Hospital), Hangzhou, 310022 China; 2 Institute of Cancer and Basic Medicine (IBMC), Chinese Academy of Sciences, Hangzhou, 310022 China; 3 Science and Technology Innovation Center Affiliated To Guangzhou, University of Traditional Chinese Medicine, Guangzhou, 510145 China; 4 Department of Neurology, Affiliated Chinese Medicine Hospital of Guangzhou Medical University, Guangzhou, 510145 China; 5 Department of Neurology, First College of Clinical Medicine Affiliated to Shandong University of Traditional Chinese Medicine, Jinan, 250014 China; 6 Department of Neurology, Peking University People’s Hospital, Beijing, 100044 China

**Keywords:** Post-stroke suicide, Stroke, Suicide, Meta-analysis, Depression

## Abstract

**Background:**

Over the past decade, increasing attention has been paid on post stroke suicide (PSS), which is one of complications of stroke. The rates of stroke and suicide are relatively high, especially in Asian populations. Thus, a deeper understanding of the prevalence and epidemiological impact of suicide after stroke is urgently needed. Clinical diagnosis and prevention of PSS are at the incipient stage, but the risk factors responsible for the occurrence of PSS in different regions and stages of the disease remain largely unknown. The present meta-analysis aimed to determine the incidence of PSS at different stages and time courses, and to identify the underlying risk factors for PSS.

**Methods:**

We systematically searched the Cochrane library, Embase, PubMed, CNKI and Web of Science databases from their inception until April 2019.The research articles reporting on the risk factor for PSS were screened and included in the meta-analysis. The data from the included studies were extracted according to the predefined criteria.

**Results:**

A total of 12 studies (*n* = 2,693,036) were included for meta-analyses. Of these studies, 7 reporting suicide prevalence were meta-analyzed. The pooled estimate of suicidal ideation rates after stroke was 12%, which could be influenced by multiple risk factors, including sex, smoking, depression, sleep disorders, previous stroke and low household income. Studies conducted in Asia demonstrated higher suicide prevalence (approximately 15%) compared to other regions. Smoking, low family income, depression, heart disease and sleep disorders were important risk factors for PSS. When compared to PSS of more than 1 year, the incidence of suicide within 1 year after stroke was more likely to be statistically significant. It was found that 4 out of every 1000 stroke survivors tended to commit suicide. The results of this meta-analysis showed that depression (OR = 2.32; *p* < 0.01) was significantly associated with suicidal ideation, regardless of stroke duration.

**Conclusion:**

PSS is one of the common complications of stroke. Despite some limitations, we successfully identified the risk factors associated with suicidal ideation after stroke. Notably, depression was significantly associated with suicidal ideation, regardless of stroke duration. Targeting this risk factor may be helpful to improve stroke patient care and prevent suicidal ideation after stroke. Future research will be carried out to assess whether suicidal ideation or thoughts and actual suicide attempts are strongly predictive of suicide deaths after stroke (Registration No. CRD42019128813).

**Supplementary Information:**

The online version contains supplementary material available at 10.1186/s12991-021-00378-8.

## Background

Stroke is recognized as one of the most devastating neurological diseases, and is characterized by a combination of physical and psychological impairments [1]. The World Health Organization (WHO) has reported that 15 million people suffer from stroke worldwide each year. Among them, 5 million die and another 5 million are permanently disabled [2]. Stroke is the second leading cause of death in the world, and this number is increasing due to population aging. Suicidal behavior is also a dramatic event that contributes to an increased risk of death and functional impairments. In the United States, approximately 32,000 individuals die by suicide each year and 5000 (14%) of them are older than 65 years of age. The rate of suicide in the elderly was 14.3 per 100,000 compared to 10.9 per 100,000 in general population [3, 4]. Suicide is one of the leading cause of death, especially in the East Asia regions [1]. In China, suicide is accounted for about 287,000 deaths every year, and the Republic of Korea has the highest incidence of suicide among the Organisation for Economic Co-operation and Development (OECD) countries [3, 5]. Previous studies have shown that physical illnesses, especially neurological disorders, can increase the risk of suicide, and the rate of suicidal ideation is higher in patients with stroke than in general population [4–6]. Consistently, several studies have demonstrated that stroke patients are at high risk for suicide, varying from 6.6 to 26.3% [2, 4–7]. Most studies suggest that this suicide risk is particularly clustered in the first few years after stroke onset [8, 9].

Although many scholars are concerned about the dangers of post-stroke suicide (PSS), the definition of this concept is still not accurate enough. According to the existing research, PSS includes suicidal ideation or thoughts, nonlethal suicide attempts, and suicide behaviors that result in death. There is an obvious difference between these three concepts, but only a few studies have reported and classified them separately. In terms of risk factors, several studies have shown that PSS is associated with depression, cognitive impairment, stroke severity and recurrent stroke [8, 10], especially in female and young adolescents [5, 11]. In addition, those who are singles, unemployed and with low education level are at 1.5–2.0-fold increased risk of having suicidal ideation among stroke survivors [12].

Given that stroke and suicide are both leading causes of death contributing to significant societal costs, it is of great importance to assess the epidemiological impact for PSS and identify the underlying risk factors [13, 14]. This study aimed to determine the incidence and underlying risk factors of PSS, especially in Asian populations, at different stages and time courses. Moreover, we sought to evaluate the incidence of actual suicide attempts (but did not lead to death) and death due to successful suicide.

## Methods

The systematic review and meta-analysis were conducted in accordance with the Preferred Reporting Items for Systematic Reviews and Meta-Analyses (PRISMA) statement. This study was registered under PROSPERO, an International Prospective Register of Systematic Reviews (Registration No. CRD42019128813).

### Search strategy

Comprehensive literature searches were carried out in April 2019. The databases such as Cochrane Stroke Group Trials Register (inception to April 2019), Medline (inception to April 2019), EMBASE (inception to April 2019) and PubMed (inception to April 2019) were searched. The search terms contained logical combinations of the keywords “stroke”, “Suicide”, “self-destruction”, “suicidal ideation after stroke” and “self-murder” (Additional file 1: Appendix SI). The China’s largest academic websites, such as Wanfang Data Knowledge Service Platform and China National Knowledge Infrastructure, were also searched. To maintain rigor, gray literature was excluded, and the literature searches were limited to peer-reviewed journals published in English or Chinese. The detailed search processes of PubMed and Embase databases are presented in Additional file 1: Appendix S1.

### Selection criteria

According to the recommendations established by the Centers for Disease Control and Prevention, suicidal ideation refers to thinking about, considering or planning suicide. This meta-analysis consists of original, observational and prospective cohort studies reporting at least one risk factor that can be analyzed, associated with PSS. The intervention study was included if baseline (pre-intervention) data were available. Studies were excluded if they contained no PSS subjects (e.g., patients with traumatic brain injury or transient ischemic attack) or lacked of primary data (e.g., review article, case study, editorial or research protocol). In case of the redundant publications reported by the same group of authors, only the study with the most exhaustive information was included.

### Data extraction

One researcher (SMZ) conducted the searches, scrutinized every titles and abstracts, identified eligible studies according to the inclusion and exclusion criteria. Irrelevant references and duplicate articles were removed, and the full text of the relevant studies were retrieved. Two researchers (SMZ and ZYQ) independently screened the full texts for eligibility. Any disagreements between these two researchers were resolved by consensus, or through arbitration by a third researcher (ZXZ) if necessary. Methodological quality of the included studies was evaluated using the Newcastle–Ottawa Scale (NOS). Critical appraisal of the quality of the included studies was performed independently by two researchers (SMZ and ARW). Disagreements between researchers were resolved by consensus, or through arbitration by a third researcher (WFZ) if necessary.

The descriptive data of the included studies, such as year of publication, country, mean age, gender ratio, sample size, time from stroke onset and methods for assessing suicidal ideation, were extracted using an Excel spreadsheet. If further information on the risk factors of suicidal ideation were needed, the corresponding author of the study was contacted to obtain the missing or unpublished data.

### Statistical analysis

Meta-analysis was conducted using Review Manager 5.3 and Stata 14.0 software. The analysis of risk factors for suicidal ideation after stroke was based on a pooled sample proportion, with related 95% confidence intervals (CIs). *I*^2^ > 75%, 50% < *I*^2^ < 75% and *I*^2^ < 50% indicated high, moderate and low degree of heterogeneity, respectively. The random effects model was selected for both medium and high heterogeneity, while fixed effects model was chosen for low heterogeneity. If more than one point factors was available in a study, the latest observation was adopted. The relationship between suicidal ideation after stroke and relevant factors was estimated by odds ratios (ORs) with 95%CIs. The meta-analysis was conducted if any risk factor data were available from at least three individual studies. Subgroup analyses were performed to assess the influence of different regions.

## Results

### Study selection and characteristics of the included studies

**Fig. 1:**
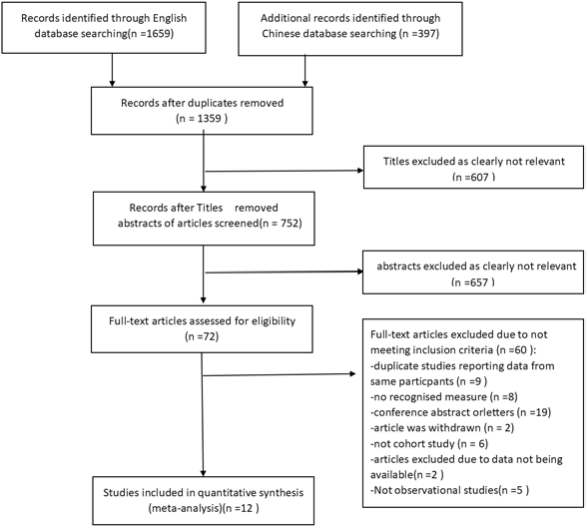
Modified PRISMA flow diagram of the included/excluded studies

The initial search yielded 2056 studies (Embase, *n* = 956; Web of Science, *n* = 441; PubMed, *n* = 235; Cochrane, *n* = 27; and CNKI, *n* = 397). After removing duplicate articles, a total of 1359 records were generated. The preliminary screening of titles and abstracts identified 72 potentially eligible studies, and the corresponding full text articles were subjected to final eligibility assessment. Finally, 12 studies (*n* = 2,693,036) were included for meta-analyses, and all studies were published since 2012 (see the PRISMA flowchart in Fig. 1 and a completed PRISMA Checklist in the second part of Additional file 1).

The median number of the included stroke patients was 1405 (range: 177–228,735). The time from stroke onset ranged from < 48 h (acute phase) to 5 years in the included studies. The follow-up period ranged from 7 days up to 12 years after stroke. Among the 12 included studies, 4 studies used the item suicidal thoughts and related depression scale to evaluate suicidal ideation; 5 studies employed Clinical Interview or questionnaire; and suicidal thought was defined accoding to the International Classification of Diseases (ICD) codes in 3 studies. All studies have a minimum NOS score of 5, indicating the good quality of the included studies. Detailed characteristics of the included studies are summarized in Table 1.

**Table 1:** Characteristics of the studies included in this meta-analysis

Study	Country	Enrolled year	Sample size	Age	Female (%)	Time after stroke	Measurement		
Santos 2012 [12]	Portugal	4.2000–6.2001	177	56.8 ± 13.1	41.2%	< 4 days	The item suicidal thoughts of the Montgomery and Asberg Depression Rating Scale	6 months	6
Jae Ho Chung 2016 [16]	Korea	2003–2008	228,735	70.1 ± 10	49.4%	Unclear	The Korean version of the World Health Organization Composite International Diagnostic Interview Short Form	12 months	6
TAKASHI YAMAUCHI 2014 [7]	Japan	1990–2010	93,027	52 ± 7.9	52.5%	0–5 years	Medicolegal examinations by licensed physicians and police investigations	10 years	7
Jin Dou 2015 [17]	China	7.2013–12.2013	281	65.2	41.45%	< 7 days	The Beck Scale for Suicide Ideation (BSI)	7 days	7
Marie Eriksson 2015 [8]	Sweden	2001–2012	220,336	> 18	48.7%	< 3 months	A suicide attempt was identified by arecord of hospital admission for or an underlying or contributing cause of death by intentional self-harm (ICD-10: X60–X84)	12 years	7
Jin Pyo Honga [18]	Korea	1.2005–12.2012	7175	62.5 ± 13	31.5%	Admission	Suicidal death was defined using the ICD-10 codes X60–X84 (intentional self-harm)	7 years	8
Eun-Young Park 2016 [19]	Korea	2006–2010	225	69.3	44.8%	Unclear	Suicidal ideation was assessed by ‘‘yes’’ or ‘‘no’’ responses to the question ‘‘Have you ever thought about suicide?’	4 years	5
Pohjasvaara 2001 [20]	Finland		486	69.9 ± 7.6	46.9%	< 3 months	Beck Depression Inventory	15 months	8
Tomor Harnod 2018 [21]	China	1.2000–12.2010	2,139,699	67	42.6%	Unclear	Followed up until a diagnosis of suicide attempt (ICD-9-CM codes E950–E959),	10 years	8
Yang 2017 [22]	China	4.2008–4.2010	2324	61.9	34.4%	< 14 days	Suicidal ideation was measured using item 3 of the Hamilton Rating Scalefor Depression	1 year	8
Altura 2016 [23]	Canada	8.2012–9.2013	204	60.1	55.7%	< 48 h	The PHQ-9、The SCID is a semistructured diagnostic interview	2 weeks	5
Wai Kwong Tang 2012 [24]	China	6.2006–9.2009	367	67 ± 107	45.2%	< 7 days	The relevant items in the Geriatric Mental State Examination-Version A	3 months	6

### Incidence of suicidal ideation in stroke survivors

**Fig. 2:**
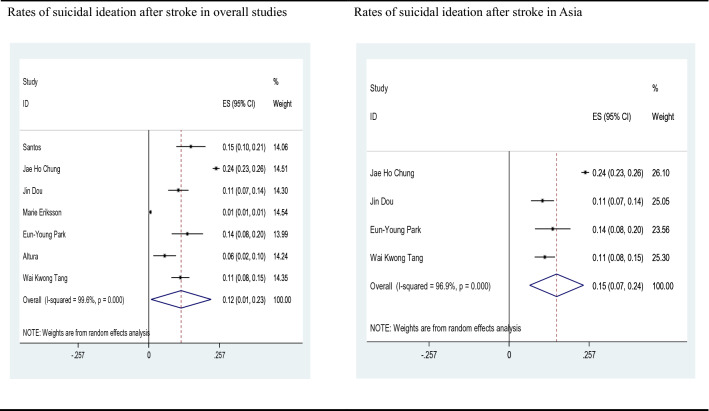
Rates of suicidal ideation among stroke survivors

**Fig. 3:**
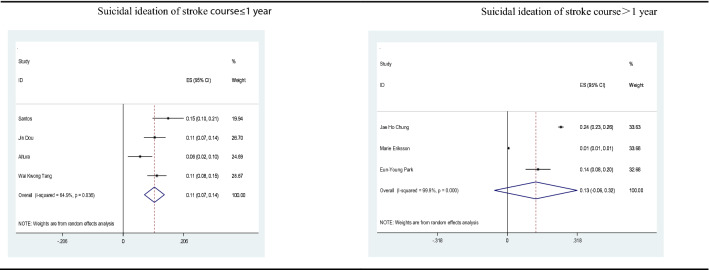
Rates of suicidal ideation among stroke survivors according to the follow-up time

**Fig. 4:**
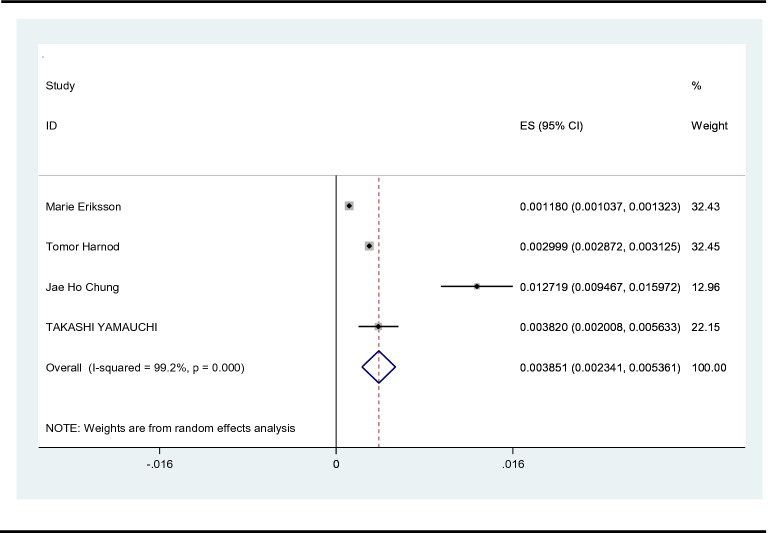
Rates of completed suicide among stroke survivors

Of the included 12 studies, 7 reported suicide prevalence and were meta-analyzed. The pooled estimate of the rate of suicidal ideation after stroke was 12%, with substantial heterogeneity between studies (*n* = 7, 12% suicidal ideation, *I*^2^ = 99.6%, 95%CI 1–23%, *p* < 0.01). The studies conducted in Asia demonstrated higher suicide prevalence (*n* = 4, 15% suicidal ideation, *I*^2^ = 96.9%, 95%CI 7–24%, *p* < 0.01) than those conducted in all regions, but a high heterogeneity was observed. Figure 2 shows the forest plot, together with cumulative estimates. It has been reported that there are increased rates of suicide after a stroke. Based on the characteristics of the included 12 studies, we analyzed the differences in the incidence and risk factors of PSS with 1 year as the boundary. According to the follow-up time, a subgroup analysis was also performed. The forest plot displayed that the incidence of suicide in less than 1 year was relatively similar to the overall study results (n = 4, 11% suicidal ideation), with moderate heterogeneity (*I*^2^ = 64.9%, 95%CI 7–14%, *p* < 0.05). Interestingly, when the suicidal ideation was assessed for more than 1 year after stroke, the incidence of suicide was not statistically significant, with high heterogeneity (*I*^2^ = 99.9%, 95%CI 6–32%, *p* = 0.178), as shown in the forest plot (Fig. 3). Furthermore, we performed a careful meta-analysis of studies reporting the number of patients who actually committed suicide. There are a total of 4 studies reporting on the incidence of actual suicide attempts and suicide deaths. Our results indicated that approximately 4 out of 1000 stroke survivors would commit suicide, with 3.85% suicide prevalence (*I*^2^ = 99.2%; 95%CI 2–5%, *p* < 0.01). The detailed findings are illustrated in Fig. 4.

### Risk factors associated with suicidal ideation after stroke

Eighteen correlates, such as sociodemographic factors (e.g., age, gender, marriage status, education level, employment status and low household income), stroke-related characteristics (e.g., location, left-sided stroke, right-sided stroke, brainstem–cerebellum and previous stroke), physical comorbid conditions (e.g., diabetes mellitus, hypertension, myocardial infarction and sleep disturbance), mental disorders (e.g., depression), and substance-related behaviors (e.g., smoking and alcohol abuse) were taken into account if the data were available from at least three individual studies. Meta-analysis of the association between these risk factors and suicidal ideation after stroke was carried out, and the results are demonstrated in Table 2. All funnel diagrams related to Table 2 are presented in Additional file 1: Appendix S2.

**Table 2:** Risk factors for suicidal ideation after stroke

Risk factors for suicidal ideation after stroke						Risk factors of suicidal ideation after stroke in Asia				
Variables	Eligible studies	Sample size	OR (95%CI)	*p* value	Heterogeneity (*I*^2^)	Eligible studies	Sample size	OR (95%CI)	*p* value	Heterogeneity (*I*^2^)
Age > 65 years	6	2,460,639	1.17 [0.99, 1.39]	0.07	82%	4	2,240,126	1.02 [0.99, 1.04]	0.26	20%
Male	9	2,463,611	1.07 [1.01, 1.13]	0.02	44%	7	2,243,098	1.05 [0.98, 1.11]	0.16	45%
Female	5	94,019	0.86 [0.60, 1.26]	0.45	19%	3	93,533	0.84 [0.49, 1.45]	0.54	32%
Left-sided stroke	4	3091	0.77 [0.53, 1.11]	0.16	0					
Right-sided stroke	3	2810	1.37 [0.99, 1.90]	0.06	0					
Brainstem–cerebellum	4	10,043	1.25 [0.57, 2.74]	0.58	70%					
Smoking	5	238,663	1.42 [1.35, 1.50]	< 0.01	15%	4	238,459	1.42 [1.35, 1.50]	< 0.01	32%
Alcohol abuse	8	2,371,850	0.73 [0.28, 1.93]	0.53	98%	3	2,370,983	2.03 [1.70, 2.42]	< 0.01	0
Married	4	231,565	0.81 [0.42, 1.55]	0.53	94%	4	231,565	0.81 [0.42, 1.55]	0.53	94%
Education	9	457,619	1.49 [0.73, 3.02]	0.27	92%	4	236,416	1.70 [0.63, 4.60]	0.3	88%
Employment	3	236,114	0.37 [0.16,0.83]	0.02	69%					
Low Household income	5	2,589,276	1.96 [1.02, 3.77]	0.04	99%	4	2,368,940	2.31 [1.17, 4.57]	0.02	98%
Depression	11	2,379,673	2.32 [1.73, 3.13]	< 0.01	96%	7	2,378,806	2.50 [1.66, 3.76]	< 0.01	98%
Diabetes mellitus	7	2,598,727	1.22 [0.98, 1.50]	0.07	81%	5	2,378,214	1.23 [0.95, 1.60]	0.12	82%
Hypertension	5	2,378,214	1.36 [0.42, 4.37]	0.6	100%	5	2,378,214	1.36 [0.42, 4.37]	0.6	100%
Myocardial infarction	5	2,367,977	1.22 [0.93, 1.61]	0.16	71%	3	2,147,155	1.23 [1.13, 1.35]	< 0.01	0
Sleeping disturbances	3	2,142,304	1.80 [1.55, 2.08]	< 0.01	0	3	2,142,304	1.80 [1.55, 2.08]	< 0.01	0
Previous stroke	8	231,146	1.55 [1.06, 2.28]	0.03	60%	4	10,147	1.31 [0.90, 1.91]	0.16	0

#### Sociodemographic factors

There were no significant effects of age (OR = 1.17; 95%CI 0.99–1.39; *p* = 0.07; *I*^2^ = 82%), female (OR = 0.86; 95%CI 0.6–1.26; *p* = 0.45; *I*^2^ = 19%), marriage status (OR = 0.81; 95%CI 0.42–1.55; *p* = 0.53; *I*^2^ = 94%) on PSS patients.Interestingly, high suicidal ideation rates were observed for males (OR = 1.07; 95%CI 1.01–1.13; *p* = 0.02; *I*^2^ = 44%)and low household income (OR = 1.96; 95%CI 1.02–3.77; *p* = 0.04); *I*^2^ = 99%, with moderate to high heterogeneity across studies. On the contrary, the rate of suicidal ideation was decreased in stroke survivors who were employed (OR = 0.37; 95%CI 0.16–0.83; *p* = 0.02; *I*^2^ = 69%). However, stroke survivors with low education level were not likely to have suicidal ideation, but the results were not statistically significant (OR = 1.49; 95%CI 0.73–3.02; *p* = 0.27; *I*^2^ = 99%). The detailed results are summarized in Table 2.

#### Stroke-related characteristics

No significant effects of left-sided stroke (OR = 0.77, 95%CI 0.53–1.11; *p* = 0.16; *I*^2^ = 0), right-sided stroke (OR = 1.37, 95%CI 0.99–1.90; *p* = 0.06; *I*^2^ = 0) and brainstem–cerebellum (OR = 1.25, 95%CI 0.57–2.74; *p* = 0.58; *I*^2^ = 70%) were found on PSS patients, with no or moderate heterogeneity across studies. Interestingly, the results showed that previous stroke was closely associated with PSS (OR = 1.55, 95%CI 1.06–2.28; *p* < 0.01; *I*^2^ = 60%). The detailed findings are presented in Table 2.

#### Physical comorbid conditions

No obvious effects of diabetes mellitus (OR = 1.22; 95%CI 0.98–1.50; *p* = 0.07; *I*^2^ = 81%), hypertension (OR = 1.36; 95%CI 0.42–4.37; *p* = 0.6; *I*^2^ = 99%) and myocardial infarction (OR = 1.22; 95%CI 0.93–1.61; *p* = 0.16; *I*^2^ = 71%) were found on PSS. The relationship between chronic diseases and PSS is unclear [16–18], and more attention should also be paid to the course, severity, and treatment effects of chronic diseases. Notably, sleep disturbance was closely associated with PSS, with no heterogeneity across studies (OR = 2.01; 95%CI 1.69–2.39; *p* < 0.01; *I*^2^ = 0). The detailed findings are shown in Table 2.

#### Mental disorders

Consistently, a remarkable increasing trend of PSS rates was observed among subjects with anxiety, with high heterogeneity across studies (OR = 2.32; 95%CI 1.73–3.13; *p* < 0.01, *I*^2^ = 96%).

#### Substance-related behaviors

There was no significant effect of alcohol abuseon PSS (OR = 0.73; 95%CI 0.28–1.93; *p* = 0.53, *I*^2^ = 98%). In contrast, stroke survivors who smoke had increased risk of PSS compared to non-smokers, with low heterogeneity across studies (OR = 1.75; 95%CI 1.35–1.50; *p* < 0.01; *I*^2^ = 15%). The detailed data can be seen in Table 2.

#### Subgroup analysis

In the subgroup analysis stratified by region, the risk factors of smoking (OR = 1.42; 95%CI 1.35–1.50; *p* < 0.01, I^2^ = 32%), low household income(OR = 2.31;95%CI 1.17–4.57; *p* = 0.02, *I*^2^ = 98%), depression (OR = 2.50; 95%CI 1.66–3.76; *p* < 0.01, *I*^2^ = 98%), myocardial infarction (OR = 1.23; 95%CI 1.13–1.35; *p* < 0.01, *I*^2^ = 0), sleep disturbance(OR = 1.80;95%CI 1.55–2.08; *p* < 0.01, *I*^2^ = 0) and alcohol abuse (OR = 2.03; 95%CI 1.70–2.42; *p* < 0.01, *I*^2^ = 0) were all closely associated with PSS in Asian populations, with 1.23–2.5-fold increases in incidence rates. Of these studied factors, the heterogeneity values were low, only those of sleep disturbance (OR = 1.80; 95%CI 1.55–2.08; *p* < 0.01, *I*^2^ = 0) and depression (OR = 2.50; 95%CI 1.66–3.76; *p* < 0.01, *I*^2^ = 98%) were high. The remaining risk factors did not significantly increase the risk of suicide in Asian populations. The detailed findings are presented in Table 2. Typically, the incidence of suicide after stroke varied over time. Therefore, a subgroup analysis was performed according to the follow-up time. Stroke survivors with older age, male, sleep disturbance and alcohol abuse were more likely to have suicidal ideation within 1 year. Besides, the risk of PSS in patients with depression was remarkably increased by 1.89–5.84-fold in less than 1 year, with moderate to high heterogeneity across studies (OR = 3.33; 95%CI 1.89–5.84; *p* < 0.01, *I*^2^ = 88%). Surprisingly, depression also increased the susceptibility of PSS, regardless of stroke duration. The remaining risk factors did not significantly increase the risk of suicide in subgroup analysis stratified by the time course of stroke. The detailed findings are demonstrated in Table 3. All funnel diagrams related to Table 3 are presented in Additional file 1: Appendix S3.

**Table 3:** Risk factors for suicidal ideation in stroke survivors according to the follow-up time

Suicidal ideation cases at ≤ 1 year post-stroke						Suicidal ideation cases at > 1 year post-stroke					
Variables	Eligible studies	Sample size	OR (95%CI)	*P* value	Heterogeneity (I^2^)	Eligible studies	Sample size		OR (95%CI)	*P* value	Heterogeneity (I^2^)
Age > 65 years						5		2,460,462	1.20 [1.01, 1.43]	0.04	85
Male	4	3149	1.08 [0.79, 1.48]	0.61	0	5		2,462,786	1.07 [1.01, 1.14]	0.02	70
Female						4		93,738	0.88 [0.58, 1.35]	0.56	39
Brainstem–cerebellum	3	2868	0.79 [0.53, 1.18]	0.25	0						
Smoking						3		231,263	1.43 [1.35, 1.51]	< 0.01	40
Alcohol abuse	3	2705	0.78 [0.32, 1.90]	0.58	72	5		2,369,145	0.78 [0.22, 2.84]	0.71	99
Education	3	662	1.54 [0.72, 3.29]	0.26	29	6		456,957	1.40 [0.59, 3.30]	0.44	94
Low Household income						4		2,588,995	1.91 [0.93, 3.94]	0.08	99
Depression	5	3353	3.33 [1.89, 5.84]	< 0.01	88	6		2,376,320	1.96 [1.33, 2.90]	< 0.01	98
Diabetes mellitus	3	2782	1.64 [0.90, 2.99]	0.1	63	4		2,595,945	1.12 [0.89, 1.41]	0.35	87
Hypertension						3		2,375,609	1.69 [0.37, 7.68]	0.5	100
Myocardial infarction						4		2,367,696	1.24 [0.92, 1.67]	0.16	78
Previous stroke	4	3149	1.30 [0.90, 1.86]	0.16	0	4		227,997	2.22 [0.86, 5.72]	0.1	82

## Discussion

Although not all stroke patients with suicidal ideation eventually commit suicide, it is understandable that suicidal thoughts can increase the risk of commit suicide in patients suffering from the sequelae of stroke. Consistent with previous studies, our meta-analysis demonstrated a high rate of suicidal ideation, accounting for approximately 12% subjects with stroke worldwide. Similar to previous studies, our results indicated the occurrence and development of PSS were influenced by multiple risk factors, including sex, smoking, depression, sleep disorders, previous stroke and low household income. Previous studies have suggested that female gender confers a substantial risk for suicidal ideation [5, 11, 12]. However, our meta-analysis revealed that male stroke survivors were more likely to commit suicide. Indeed, this trend was consistent with the published studies showing that the incidence rates of suicide were higher in men than in women, regardless of stroke status [25].

Smoking, low family income, depression, heart disease and sleep disorders were the important risk factors for PSS. According to the results of our meta-analysis, stroke-related clinical factors, such as smoking, depression, sleep disturbance andrecurrent stroke, appear to play a critical role in the increased rates of PSS. Recurrent stroke, depression and sleep disturbance raised the PSS rates approximately twice, while older age, men, smoking and depression were closely related to PSS for more than 1 year. The main risk factors for PSS were previous stroke and depression [26], but only the predictive value of depression was confirmed [12]. Depression after stroke, together with suicidal ideation, could worsen stroke outcomes and life expectancy, by affecting treatment adherence [27, 28]. The results of this meta-analysis showed that depression was significantly associated with suicidal ideation, regardless of stroke duration. Therefore, prevention and early intervention of depression after stroke should be an essential part of stroke rehabilitation, to reduce the risk of PSS-related behaviors, even though the biological mechanisms underlying PSS remain largely unclarified [19, 29].

Contradictory results have been reported on the location and laterality of PSS [30, 31], and we did not observe a clear trend on this point as well. Both socioeconomic and clinical factors have been shown to increase the risk for suicide [8, 32]. From the clinical points of view, it can be speculated that Asian populations are more embarrassed towards stroke disability. Economic level and education level affect people's acceptance of limb dysfunction. Compared to European countries, most Asian regions are underdeveloped. Insurance systems in Asia are not universal, and with poor socio-economic development, stroke patients will have to bear a heavier financial burden and greater psychological pressure of not being able to provide income for their families. This may partly explain the higher risk of suicidal ideation in Asia. Epidemiological evidence has indicated that the incidence of PSS is higher in Asian populations, accounting for approximately 15%. In this study, we sought to explore the difference in risk factors between PSS in Asia and all regions. Regardless of the whole study populations or Asian subgroup, our findings showed that low household income nearly doubled the incidence of PSS. This suggests that stroke survivors with low socioeconomic status may be more susceptible to commit suicide [33]. Considering that delayed suicidal plans were associated with poor social support [12], the improvement of medical care and insurance investment for low-income groups can potentially help to prevent suicidal ideation, especially in Asian populations.

A considerable proportion of stroke survivors attempted to commit suicide within 1 year [34, 35], and the risk decreased sharply after 5 years [7]. Other studies also found that the risk of suicide was particularly high in Europe within the first 2 years after stroke [20, 36]. Similar results were observed in the present meta-analysis. In fact, compared to PSS more than 1 year, the incidence of suicide within 1 year after stroke was more likely to be statistically significant. Thus, more efforts should be focused on this point, which can effectively help to prevent PSS.

In addition, among the tested sociodemographic characteristics, ‘being employed’was identified as a protective factor for PSS, which is in line with our findings that low household income is a risk factor for PSS. Our findings revealed that 4 out of every 1,000 stroke survivors committed suicide, which was the most innovative aspect of this study. Given that a previous suicide attempt is a strong predictor for future suicide attempt [37, 38], targeted treatment for patients with the above-mentioned risk factors can help to improve the overall prognosis of stroke. For instance, stroke survivors who smoke and jobless as well as those with low household income, depression, sleep disorders and recurrent stroke, should be specifically targeted for suicide prevention.

Nevertheless, this meta-analysis has some limitations; hence, the results should be interpreted with caution. First, we found moderate to high heterogeneity across some studies for assessing the rate and risk factors of PSS. Consistent with other studies, such phenomenon might be attributed to some methodological differences in terms of inclusion criteria, stroke severity, assessment time points, study regions and screening measures for suicidal ideation.

Second, the diverse selection of methods for evaluating suicidal ideation partly leads to a high study heterogeneity. In particular, only few studies have examined PSS with the purposively developed measure, such as the Beck Scale for Suicidal Ideation (BSI). The majority of studies acquired the information of suicidal ideation from the scales for exploring depression. Moreover, only a limited number of included studies reported on the detailed data of risk factors, such as alcohol consumption, smoking, diabetes severity, hypertension grades and heart disease types, which in turn limits the actual confidence of estimates for some variables.

Third, numerous studies excluded stroke patients with communication or cognitive impairment, and no conclusion can be drawn from these studies. Although the limitation regarding cognition or communication impairment is common in many studies, it is necessary to pay some attention on patients with less-severe illness across the study populations.

Finally, comprehensive literature search and contacting corresponding authors for additional data can help us to minimize the possibility of bias, but it is impossible to fully exclude the potential bias. Besides, we are focusing on Asian patients who committed suicide after stroke. Therefore, it is essential to retrieve Chinese academic articles. However, there is a lack of articles written in Chinese about the risk factors of PSS. We also hope to encourage Chinese doctors to explore this aspect through our meta-analysis.

## Conclusion

PSS is one of the common complications of stroke. A deeper understanding of risk factors for suicidal ideation may not only enable healthcare workers to realize this ‘invisible handicap’, but also facilitate the implementation of new prevention strategies. Despite several limitations, this meta-analysis identified a number of risk factors for suicidal ideation in stroke survivors. Considering the high incidence of PSS within 1 year, new interventions should be designed to reduce suicide risk in stroke survivors by focusing on the multiple risk factors identified during this time frame.

## Supplementary Information

**Additional file 1.** EDITORIAL CERTIFICATE: Proof of language polish for this paper. PRISMA 2009 Checklist: The PRISMA Checklist of our meta-analysis. Appendix 1: An example of how we search on Pubmed and Embase. Appendix 2: Funnel plots for meta-analyses in Table 2. Appendix 3: Funnel plots for meta-analyses in Table 3.

## Data Availability

All data generated or analyzed during this study are included in this article. All data are fully available without restriction.

## Abbreviations

PSS: Post-Stroke Suicide
NOS: Newcastle–Ottawa Scale
PRISMA: Preferred Reporting Items for Systematic Reviews and Meta-Analyses
PHQ-9: Patient Health Questionnaire-9
SCID: Structured Clinical Interview for DSM
BSI: The Beck Scale for Suicide Ideation
