# Supplementary material for: Meta-analysis of risk factors associated with suicidal ideation after stroke
Source: Ann Gen Psychiatry. 2022 Jan 5;21:1. doi: 10.1186/s12991-021-00378-8 (PMC8734070; doi:10.1186/s12991-021-00378-8)
Supplement: Supplementary file 1 — Additional file 1. EDITORIAL CERTIFICATE: Proof of language polish for this paper. PRISMA 2009 Checklist: The PRISMA Checklist of our meta-analysis. Appendix 1: An example of how we search on Pubmed and Embase. Appendix 2: Funnel plots for meta-analyses in Table 2. Appendix 3: Funnel plots for meta-analyses in Table 3. [file 12991_2021_378_MOESM1_ESM.pdf]

EDITSPRINGS

EDITORIAL CERTIFICATE

This is to certify that the manuscript detailed below was edited by one or more of our highly qualified, native English speakers at EditSprings, to assure compliance with Anglophone academic standards in terms of style, punctuation, grammar, and spelling.

Manuscript title:

Meta-analysis of risk factors associated with suicidal ideation after stroke

Authors:

Shuangmei Zhang;An rong Wang;Weifeng Zhu;Zhaoyang Qiu;Zhao xu Zhang

Date Issued:

Nov 05 2021

Certificate Number:

ES-202002231104278759

This certificate can be verified on https://www.editsprings.cn/QueryCertificate.html EditSprings hereby certifies that neither content nor the author's intentions were altered in any way during the editing process. Documents in receipt of this certification should be ready for publication as far as style and language are concerned, provided that the author(s) accepted our suggestions and changes (which remains their right and responsibility).

EditSprings offers a wide range of editing, translation, for researchers and publishers across the world. Our highly skilled editors are all established academics based in Anglophone Higher Education institutions across the world (U.K., U.S.A., Canada, Australia, and elsewhere), are experts in their respective fields, and are qualified to edit research papers authored by non-Anglophone scholars.

EDITORIAL CERTIFICATE

This document certifies that the manuscript listed below was edited for proper English language, grammar, punctuation, spelling, and overall style by one or more of the highly qualified native English speaking editors at EditSprings.

Manuscript title:

Meta-analysis of risk factors associated with suicidal ideation after stroke

Authors:

Shuangmei Zhang , An rong Wang , Weifeng Zhu , Zhaoyang Qiu , Zhao xu Zhang

Date Issued:

Jul 13 2021

Certificate Number:

ES-2020022311104278759

This certificate can be verified on www.editsprings.com/query.asp. This document certifies that the manuscript listedabove was edited for proper English language, grammar,punctuation, spelling, and overall style by one or more of thehighly qualified native English speaking editors at EditSprings. Neither the research content nor the authors' intentionswere altered in any way during the editing process. Documents receiving this certification should be English-ready forpublication; however, the author has the ability to accept or reject our suggestions and changes.

EditSprings provides a range of editing, translation and manuscript services for researchers and publishersaround the world. Our top-quality PhD editors are all native English speakers from famous institutions crossthe U.S., Britain, Canada and so on.Our editors come from nearly every research field and possess thehighest qualifications to edit research manuscripts written by non-native English speakers.

EditSprings

EDITORIAL CERTIFICATE

This document certifies that the manuscript listed below was edited for proper English language, grammar, punctuation, spelling, and overall style by one or more of the highly qualified native English speaking editors at EditSprings.

Manuscript title:

Meta-analysis of risk factors associated with suicidal ideation after stroke: A focus on Asian populations

Authors:

Shuangmei Zhang,Zhaoyang Qiu,Weifeng Zhu,An rong Wang,
Zhao xu Zhang

Date Issued:

Mar 11 2020

Certificate Number:

ES-2020022311O4278759

This certificate can be verified on www.editsprings.com/query.asp. This document certifies that the manuscript listed above was edited for proper English language, grammar, punctuation, spelling, and overall style by one or more of the highly qualified native English speaking editors at EditSprings. Neither the research content nor the authors' intentions were altered in any way during the editing process. Documents receiving this certification should be English-ready for publication; however, the author has the ability to accept or reject our suggestions and changes.

EditSprings provides a range of editing, translation and manuscript services for researchers and publishers around the world. Our top-quality PhD editors are all native English speakers from famous institutions cross the U.S., Britain, Canada and so on. Our editors come from nearly every research field and possess the highest qualifications to edit research manuscripts written by non-native English speakers.

PRISMA 2009 Checklist

Section/topic	#	Checklist item	Reported on page #
**TITLE**			P1-Title
Title	1	Identify the report as a systematic review, meta-analysis, or both.	
**ABSTRACT**			
Structured summary	2	Provide a structured summary including, as applicable: background; objectives; data sources; study eligibility criteria, participants, and interventions; study appraisal and synthesis methods; results; limitations; conclusions and implications of key findings; systematic review registration number.	P2-3 Abstract , Methods paragraph 1
**INTRODUCTION**			
Rationale	3	Describe the rationale for the review in the context of what is already known.	P2 Background paragraph 1
Objectives	4	Provide an explicit statement of questions being addressed with reference to participants, interventions, comparisons, outcomes, and study design (PICOS).	P2-3 Background paragraph 2-3
**METHODS**			
Protocol and registration	5	Indicate if a review protocol exists, if and where it can be accessed (e.g., Web address), and, if available, provide registration information including registration number.	P3 Methods paragraph 1
Eligibility criteria	6	Specify study characteristics (e.g., PICOS, length of follow-up) and report characteristics (e.g., years considered, language, publication status) used as criteria for eligibility, giving rationale.	P3 Methods paragraph 2
Information sources	7	Describe all information sources (e.g., databases with dates of coverage, contact with study authors to identify additional studies) in the search and date last searched.	P3 Methods paragraph 2
Search	8	Present full electronic search strategy for at least one database, including any limits used, such that it could be repeated.	P3 Methods paragraph 2,Appendix
Study selection	9	State the process for selecting studies (i.e., screening, eligibility, included in systematic review, and, if applicable, included in the meta-analysis).	P3 Procedure
Data collection process	10	Describe method of data extraction from reports (e.g., piloted forms, independently, in duplicate) and any processes for obtaining and confirming data from investigators.	P3 Procedure

Data items	11	List and define all variables for which data were sought (e.g., PICOS, funding sources) and any assumptions and simplifications made.	P3<br>Procedure
Risk of bias in individual studies	12	Describe methods used for assessing risk of bias of individual studies (including specification of whether this was done at the study or outcome level), and how this information is to be used in any data synthesis.	P3-4<br>Methods
Summary measures	13	State the principal summary measures (e.g., risk ratio, difference in means).	P4<br>Statistical analysis paragraph 1
Synthesis of results	14	Describe the methods of handling data and combining results of studies, if done, including measures of consistency (e.g., I^2) for each meta-analysis.	P4<br>Statistical analysis<br>paragraph 1

Section/topic	#	Checklist item	Reported on page #
Risk of bias across studies	15	Specify any assessment of risk of bias that may affect the cumulative evidence (e.g., publication bias, selective reporting within studies).	P3-4<br>Methods,Statistical analysis
Additional analyses	16	Describe methods of additional analyses (e.g., sensitivity or subgroup analyses, meta-regression), if done, indicating which were pre-specified.	P4<br>Statistical analysis<br>paragraph 1
**RESULTS**			
Study selection	17	Give numbers of studies screened, assessed for eligibility, and included in the review, with reasons for exclusions at each stage, ideally with a flow diagram.	P4 Results paragraph 1,Figure 1
Study characteristics	18	For each study, present characteristics for which data were extracted (e.g., study size, PICOS, follow-up period) and provide the citations.	P4 -6Results paragraph 1-9,Table 1.
Risk of bias within studies	19	Present data on risk of bias of each study and, if available, any outcome level assessment (see item 12).	P4-6 Results paragraph 1-9,Appendix
Results of individual studies	20	For all outcomes considered (benefits or harms), present, for each study: (a) simple summary data for each intervention group (b) effect estimates and confidence intervals, ideally with a forest plot.	P4-6 Results paragraph 1-9,,Figure 2-4<br>Table 2-3
Synthesis of results	21	Present results of each meta-analysis done, including confidence intervals and measures of consistency.	P4-6 Results paragraph 1-9,,Figure 2-4<br>Table 2-3

Risk of bias across studies	22	Present results of any assessment of risk of bias across studies (see Item 15).	P4-6 Results paragraph 1-9,,Figure 2-4<br>Table 1-3
Additional analysis	23	Give results of additional analyses, if done (e.g., sensitivity or subgroup analyses, meta-regression [see Item 16]).	P4-6 Results paragraph 9,,Figure 4<br>Table 2-3
**DISCUSSION**			
Summary of evidence	24	Summarize the main findings including the strength of evidence for each main outcome; consider their relevance to key groups (e.g., healthcare providers, users, and policy makers).	P6-7 Discussion<br>paragraph 1-5
Limitations	25	Discuss limitations at study and outcome level (e.g., risk of bias), and at review-level (e.g., incomplete retrieval of identified research, reporting bias).	P7 Discussion<br>paragraph 6-9
Conclusions	26	Provide a general interpretation of the results in the context of other evidence, and implications for future research.	P7 Conclusions<br>paragraph 1
**FUNDING**			
Funding	27	Describe sources of funding for the systematic review and other support (e.g., supply of data); role of funders for the systematic review.	P8 Funding<br>paragraph 1

From: Moher D, Liberati A, Tetzlaff J, Altman DG, The PRISMA Group (2009). Preferred Reporting Items for Systematic Reviews and Meta-Analyses: The PRISMA Statement. PLoS Med 6(7): e1000097. doi:10.1371/journal.pmed1000097

For more information, visit: **www.prisma-statement.org**.

Appendix 1

PUBMED :("Suicide"[Mesh]) AND (((((("Stroke"[Mesh] OR (Strokes[TIAB]) OR (Cerebrovascular Accident[TIAB]) OR (Cerebrovascular Accidents[TIAB]) OR (CVA (Cerebrovascular Accident)[TIAB]) OR (CVAs (Cerebrovascular Accident)[TIAB]) OR (Cerebrovascular Apoplexy[Title/Abstract]) OR (Apoplexy,Cerebrovascular[Title/Abstract]) OR (Vascular Accident,Brain[Title/Abstract]) OR (Brain Vascular Accident[TIAB]) OR (Brain Vascular Accidents[TIAB]) OR (Vascular Accidents,Brain[TIAB]) OR (Cerebrovascular Stroke[TIAB]) OR (Cerebrovascular Strokes[TIAB]))) OR (Strokes, Cerebrovascular[TIAB]) OR (Stroke, Cerebrovascular[Title/Abstract]) OR (Apoplexy[Title/Abstract]) OR (Stroke, Cerebral[Title/Abstract]) OR (Strokes, Cerebral[TIAB]) OR (Stroke, Acute[TIAB]) OR (Acute Stroke[TIAB]) OR (Acute Strokes[TIAB]) OR (Strokes, Acute[TIAB])OR (Cerebrovascular Accident, Acute[TIAB]) OR (Acute Cerebrovascular Accident[TIAB]) OR (Acute Cerebrovascular Accidents[TIAB]) OR (Cerebrovascular Accidents, Acute[TIAB]))))))

Embase®

Embase Session Results

No.	Query	Results
#5	**#3** OR **#4**	956
#4	**#1** AND **#2**	823
#3	**'suicidal ideation after stroke'** OR (**suicidal** AND **ideation** AND **after** AND (**'stroke'**/exp OR **stroke**)) OR **'suicide after stroke'** OR ((**'suicide'**/exp OR **suicide**) AND **after** AND (**'stroke'**/exp OR **stroke**))	369
#2	**suicide** OR **'suicide'**/exp	106,684
#1	**'cerebrovascular accident'**/exp	306,486

Appendix2

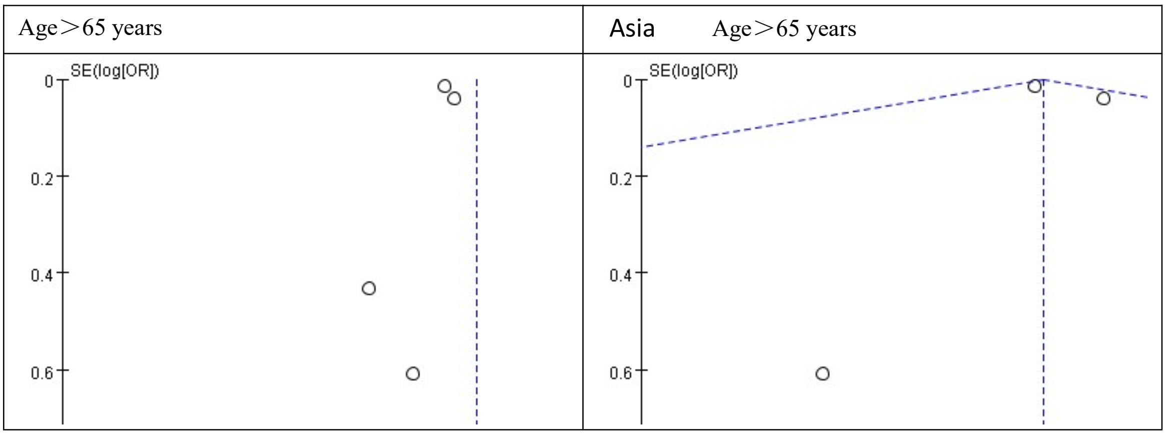

Male

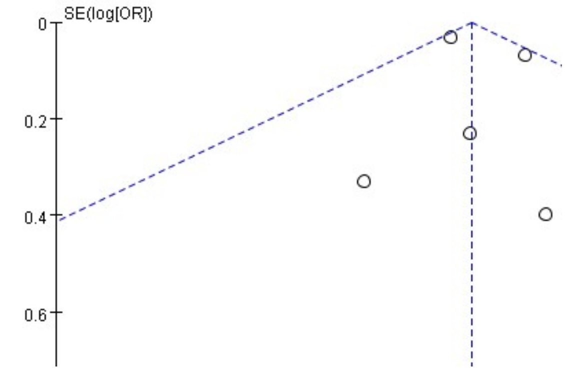

Asia Male

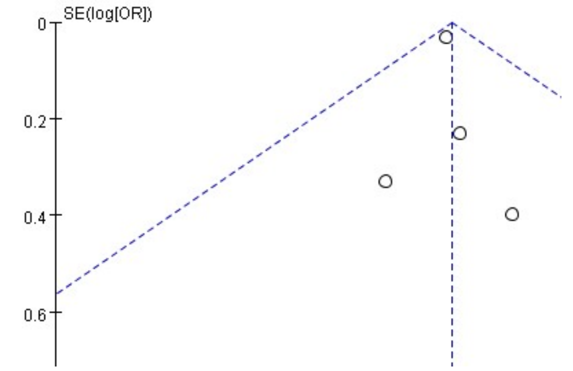

Female

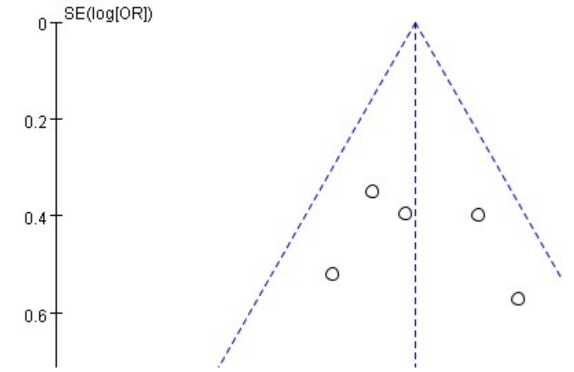

Asia Female

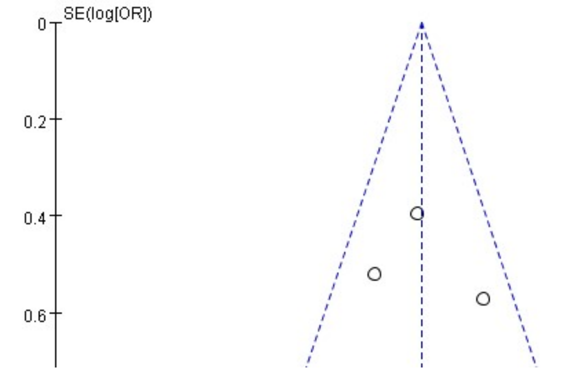

Left-sided stroke

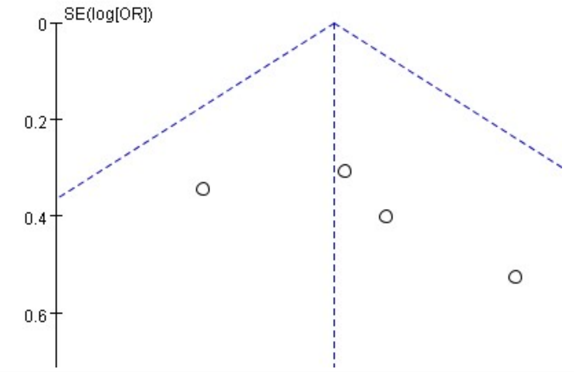

Right-sided stroke

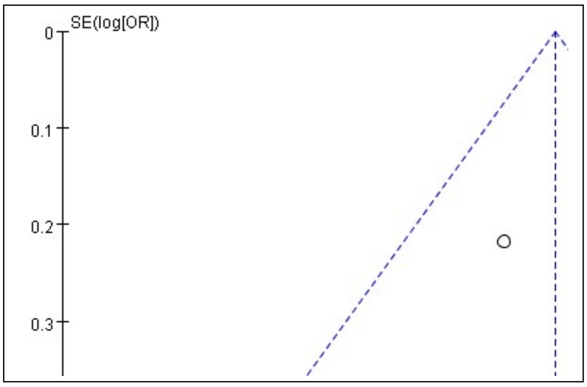

Brainstem-cerebellum

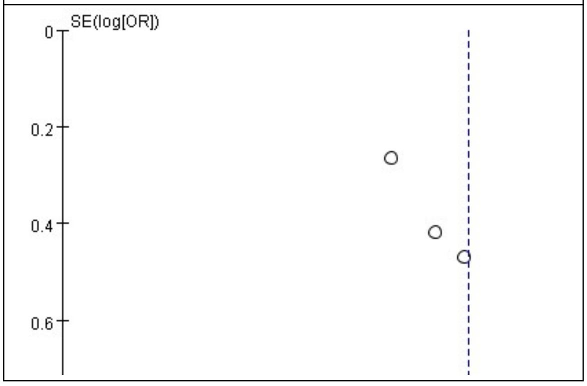

Smoker	Asia Smoker

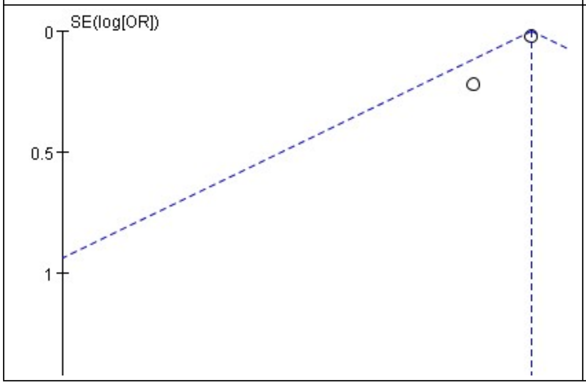

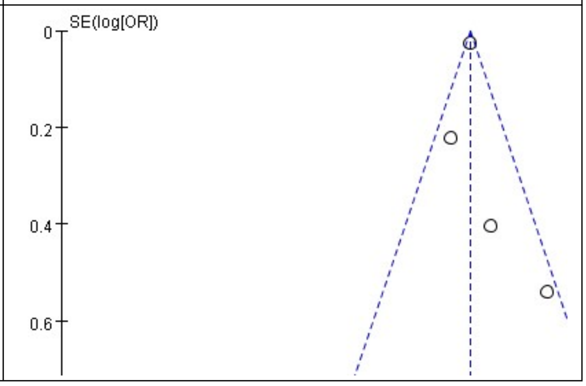

Alcohol abuse	Asia Alcohol abuse

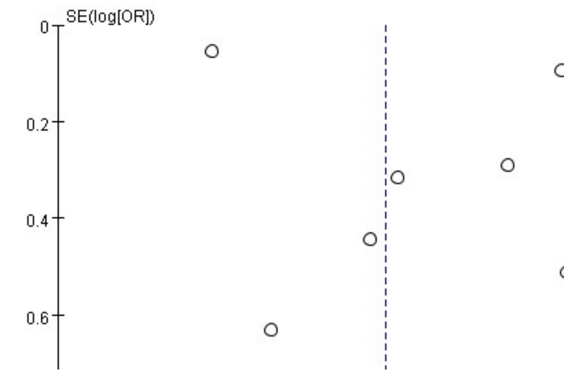

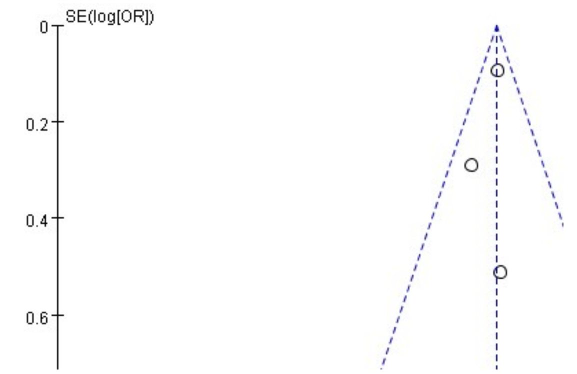

Married	Asia Married
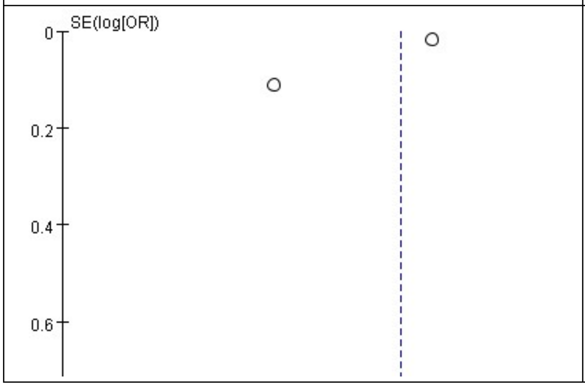	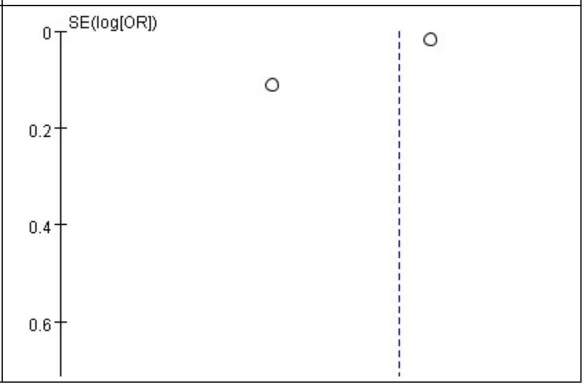

Education	Asia Education
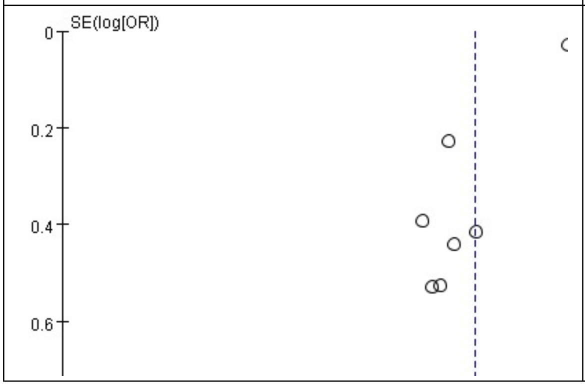	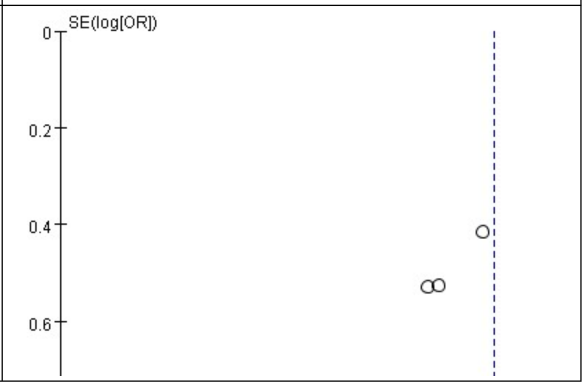

Employment

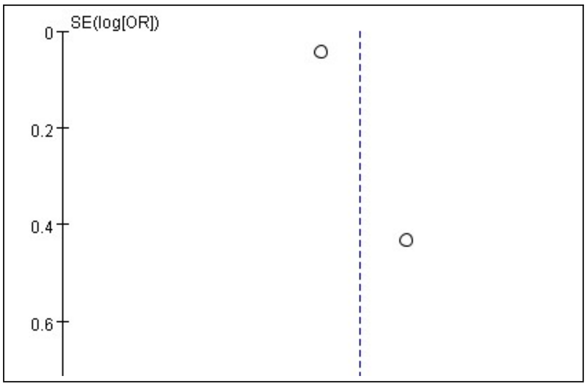

Low Household income

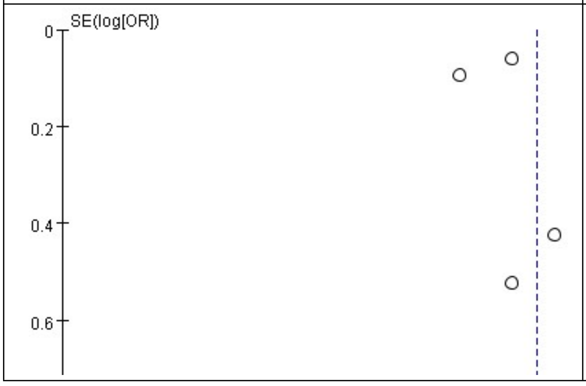

Asia Low Household income

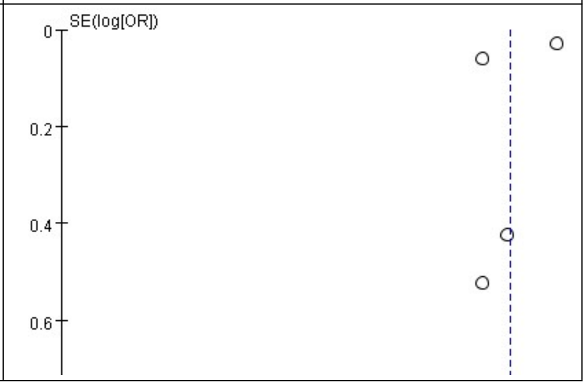

Depression

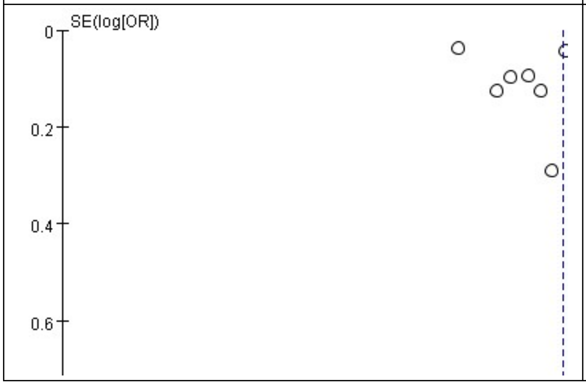

Asia Depression

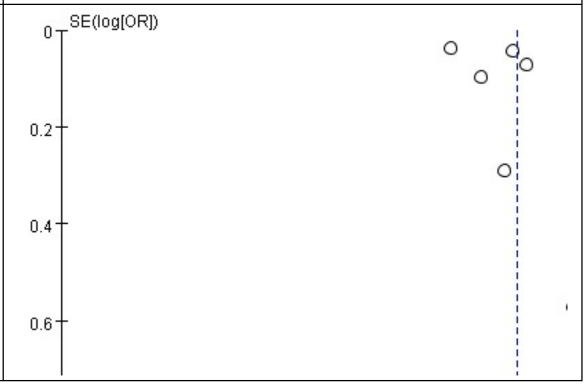

Diabetes mellitus

Asia Diabetes mellitus

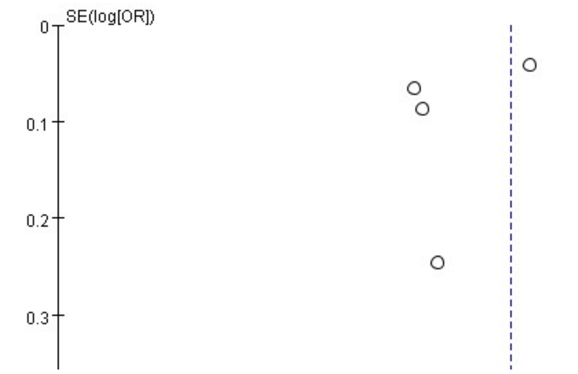

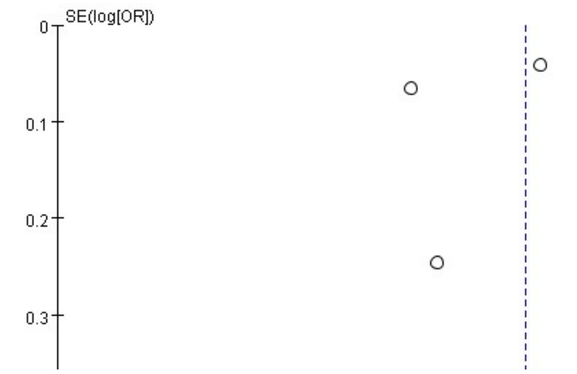

Hypertension	Asia Hypertension

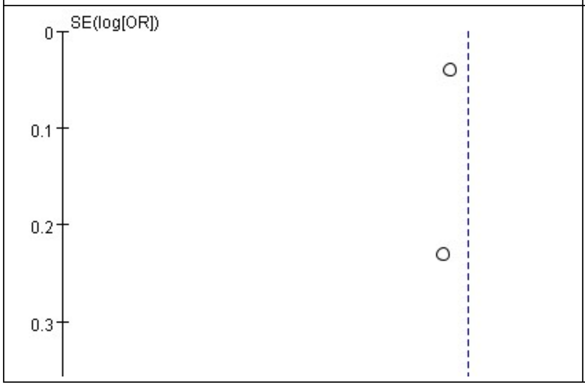

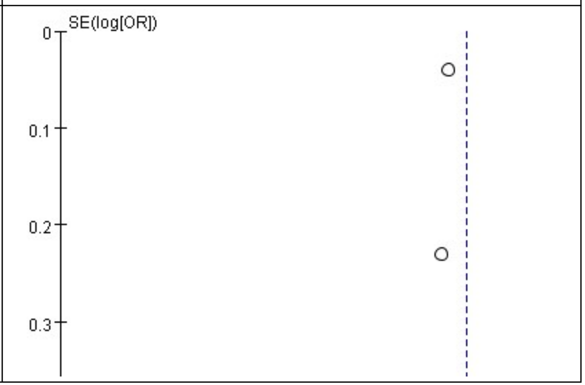

Myocardial infarction	Asia Myocardial infarction

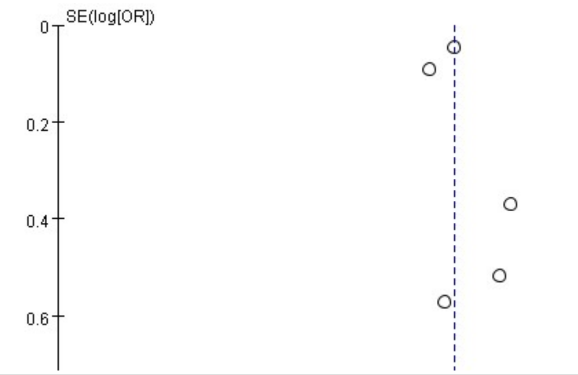

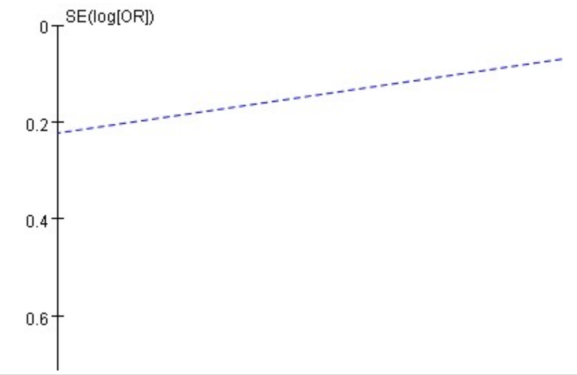

Sleeping disturbances	Asia Sleeping disturbances

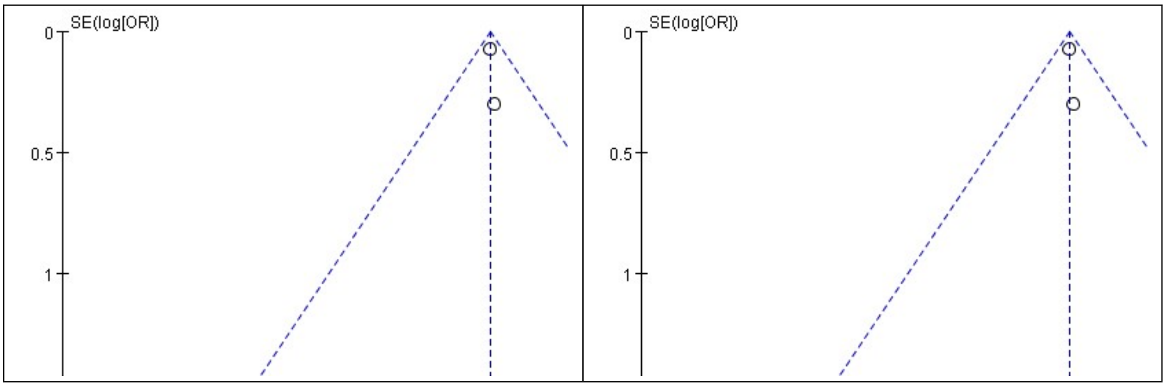

Previous stroke

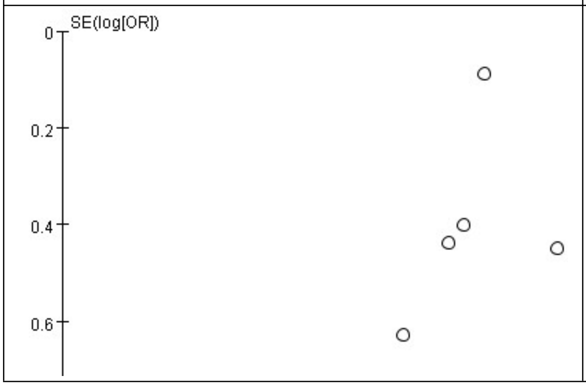

Asia Previous stroke

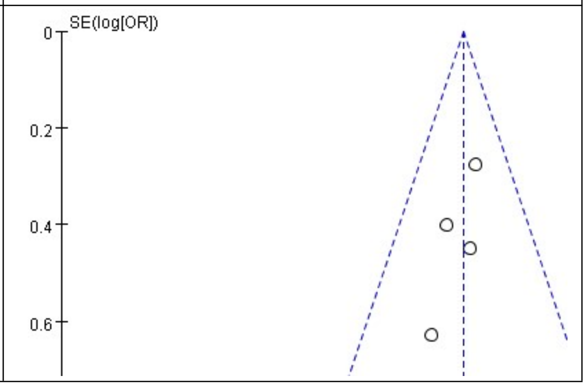

Appendix3

Age＞65 years	
suicidal ideation of stroke course≤1 year	suicidal ideation of stroke course＞1 year
	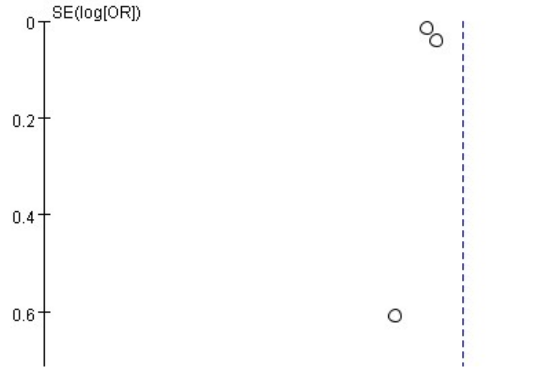

Male	
suicidal ideation of stroke course≤1 year	suicidal ideation of stroke course＞1 year
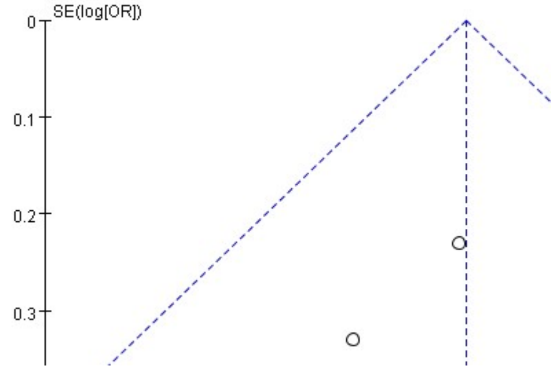	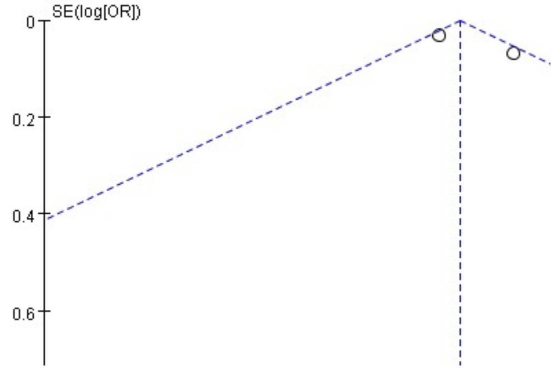

Female	
suicidal ideation of stroke course≤1 year	suicidal ideation of stroke course＞1 year
	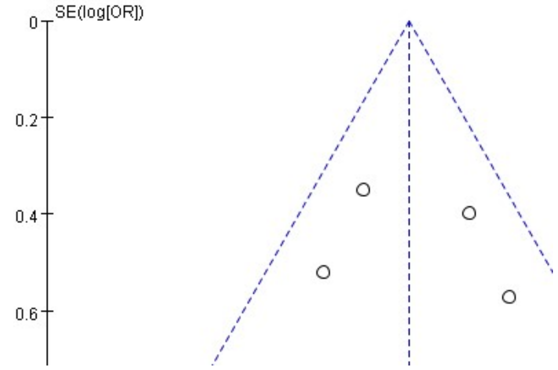

Brainstem-cerebellum	
suicidal ideation of stroke course≤1 year	suicidal ideation of stroke course＞1 year
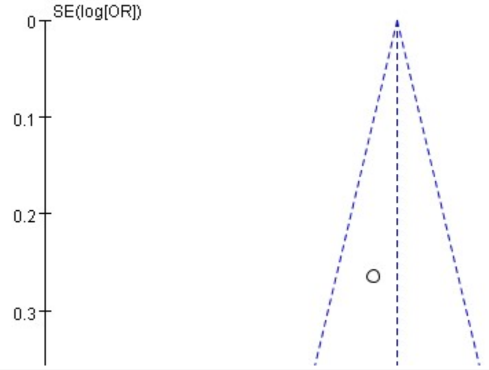	

Smoker	
suicidal ideation of stroke course≤1 year	suicidal ideation of stroke course＞1 year

<table>
<tr><td></td><td>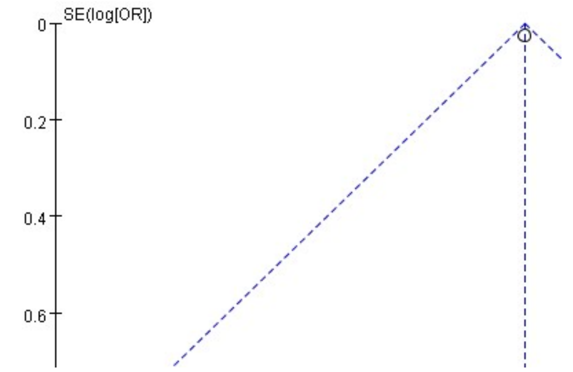
</td></tr>
</table>

<table>
<tr><td colspan="2">Alcohol abuse</td></tr>
<tr><td>suicidal ideation of stroke course≤1 year</td><td>suicidal ideation of stroke course＞1 year</td></tr>
<tr><td>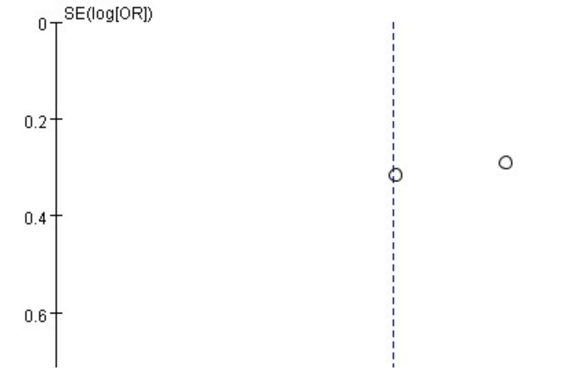
</td><td>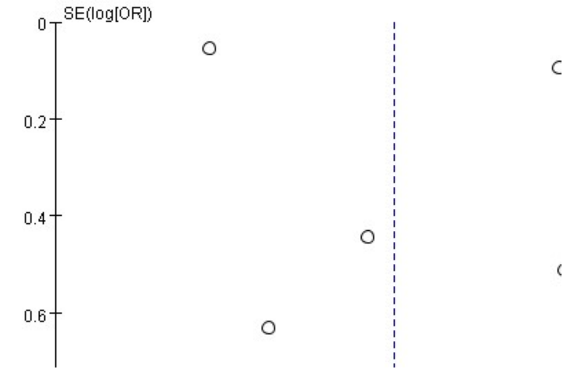
</td></tr>
</table>

<table>
<tr><td colspan="2">Education</td></tr>
<tr><td>suicidal ideation of stroke course≤1 year</td><td>suicidal ideation of stroke course＞1 year</td></tr>
</table>

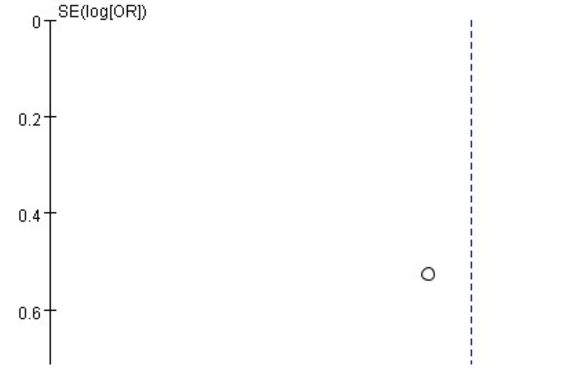	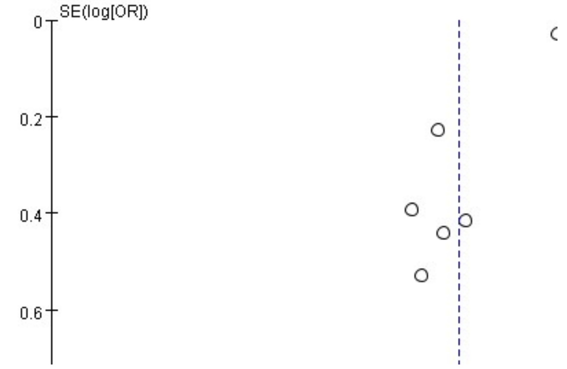

Low Household income	
suicidal ideation of stroke course≤1 year	suicidal ideation of stroke course＞1 year
	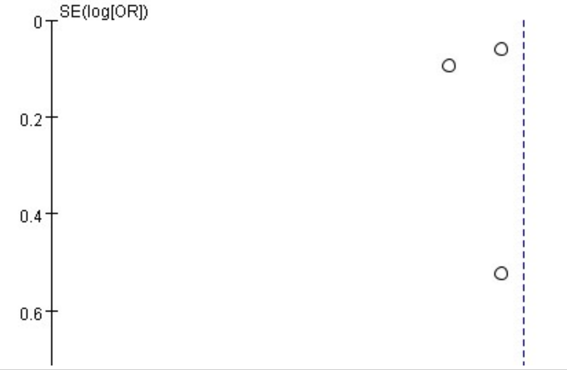

Depression

suicidal ideation of stroke course≤1 year	suicidal ideation of stroke course＞1 year
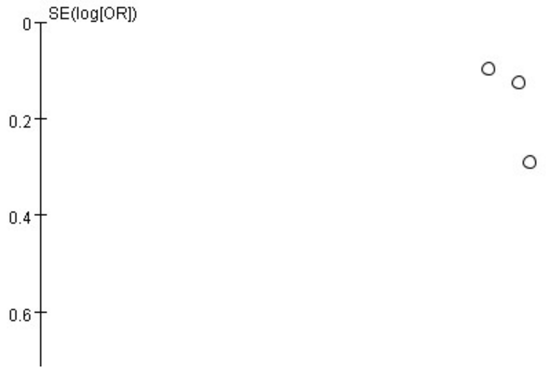	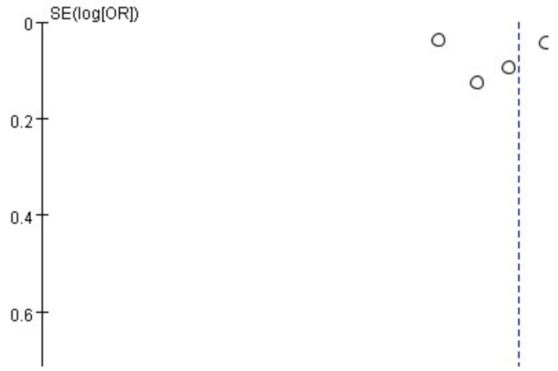

Diabetes mellitus	
suicidal ideation of stroke course≤1 year	suicidal ideation of stroke course＞1 year
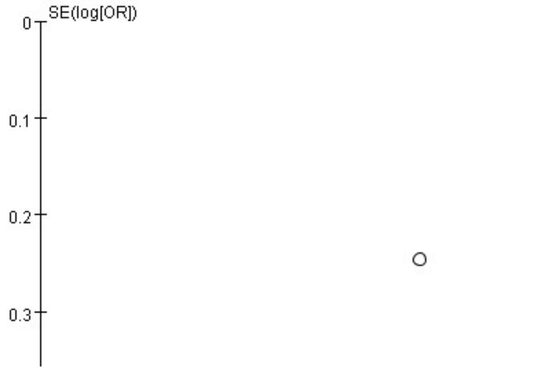	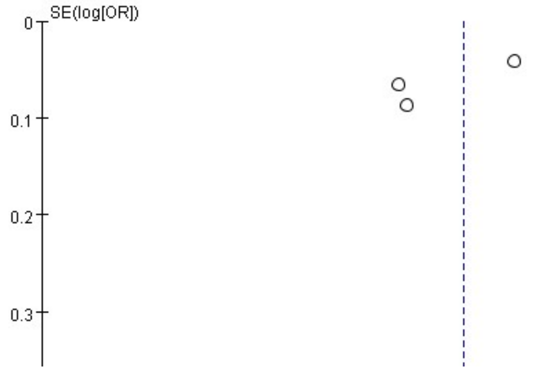

Hypertension	
suicidal ideation of stroke course≤1 year	suicidal ideation of stroke course＞1 year
	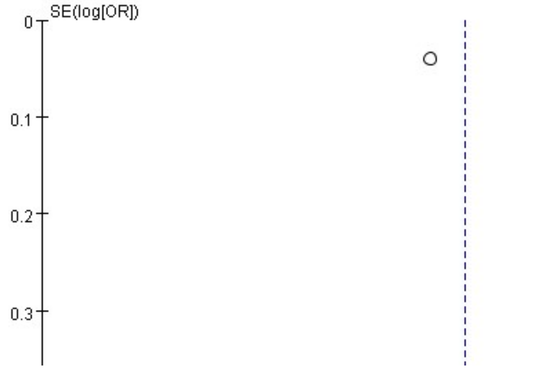

<table>
<tr><td colspan="2">Myocardial infarction</td></tr>
<tr><td>suicidal ideation of stroke course≤1 year</td><td>suicidal ideation of stroke course＞1 year</td></tr>
<tr><td></td><td>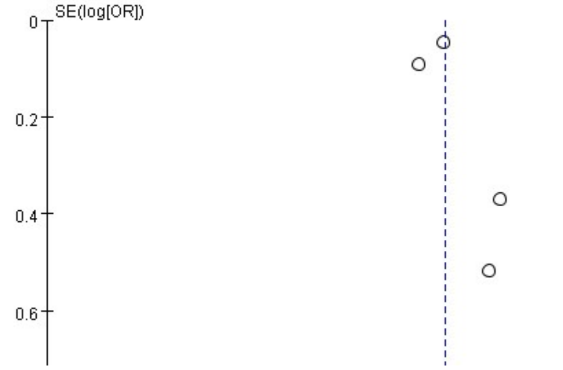</td></tr>
</table>

<table>
<tr><td colspan="2">Previous stroke</td></tr>
<tr><td>suicidal ideation of stroke course≤1 year</td><td>suicidal ideation of stroke course＞1 year</td></tr>
<tr><td>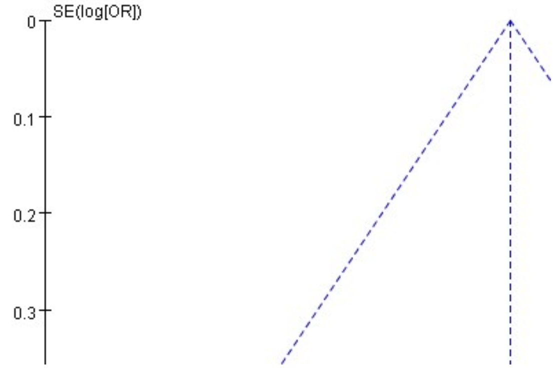</td><td>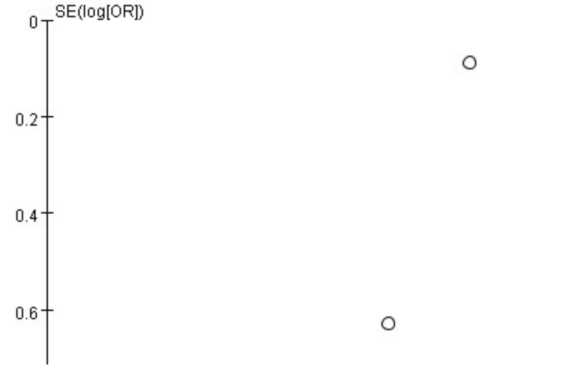</td></tr>
</table>
